# Three May Be Better Than Two: A Proposal for Metformin Addition to PI3K/Akt Inhibitor-antiandrogen Combination in Castration-resistant Prostate Cancer

**DOI:** 10.7759/cureus.3403

**Published:** 2018-10-02

**Authors:** Mohamed Islam Delma

**Affiliations:** 1 Endocrinology, Colonel Chaabani Hospital, El Menia, DZA

**Keywords:** castration-resistant prostate cancer, pi3k/akt inhibitors, metformin

## Abstract

Prostate cancer is a prevalent malignant disease. Castration-resistant prostate cancer (CRPC) is a poor prognosis form that develops upon resistance to first-line androgen deprivation therapy. Intensive research is ongoing to find efficient therapeutics for this refractory state. Actually, the combination of PI3K/Akt inhibitors with new-generation antiandrogens is among the most promising therapeutic schemes, although not yet at the optimal level. Metformin effects on prostate cancer, notably its therapeutic targets shared with antiandrogens and/or PI3K/Akt inhibitors, are reviewed in this article. From that, the hypothesis of PI3K/Akt-antiandrogens dual blockade optimization by metformin addition in CRPC will be deduced.

## Introduction and background

The incidence of prostate cancer is approximately 1.6 million cases per year, making it among the most commonly diagnosed cancers in men [1]. Androgen deprivation therapy has been the mainstay of palliative treatment in advanced and metastatic prostate cancer for many years. It is also increasingly used in patients with localized disease [2]. Unfortunately, within approximately five years of follow-up, 10%–20% of treated patients become refractory to hormonal therapy, a condition called castration-resistant cancer prostate (CRPC) [3]. It is defined by the Canadian Urology Association as disease progression despite castrate levels of testosterone and may present as either a continuous rise in serum prostate-specific antigen (PSA) levels, the progression of a pre-existing disease, and/or the appearance of new metastases [4].

Among the mechanisms incriminated in the development of CRPC, some are related to genetic alterations affecting the androgen receptor (AR) activity, such as a mutation in the ligand binding domain, an amplification of the AR gene, and an expression of splice variants. Others concern the intracellular upregulation of genes responsible for converting adrenal androgens to their more potent form, dihydrotestosterone [5].

The actual therapeutic panel for this condition includes new generation antiandrogens, such as abiraterone acetate and enzalutamide, chemotherapy, and bone-targeted therapy with radium-223 [4]. Given their actual limited efficacy, the guidelines encourage the enrollment of patients into clinical trials in search of more efficient drugs or therapeutic schemes.

This paper reviews the outcomes of the inclusion of Akt/PI3K pathway inhibitors in CRPC clinical trials, as a monotherapy and in combination with androgen inhibitors. After that, it discussed the possibility of potentiating the aforementioned combination.

## Review

PI3K/Akt pathway

The PI3K/Akt pathway has been well illustrated by Hemmings and Restuccia in their review based on original research papers [6]. The cascade begins with the activation of phosphatidylinositol 3-kinase (PI3K) by growth factor receptors. Activated PI3K induces the phosphorylation of phosphatidylinositol (4,5)-bisphosphate (PIP2) to phosphatidylinositol (3,4,5)-trisphosphate (PIP3) at the inner side of the plasma membrane. Protein kinase B (Akt) interaction with these phospholipids causes its translocation to the inner membrane, where protein 3-phosphoinositide-dependent protein kinase-1 (PDK1) is located. Akt binds to PIP3 at the plasma membrane, allowing PDK1 to access and phosphorylate Akt at residue T308, leading to its partial activation. Akt is responsible for the activation of many effectors, among them the mammalian target of rapamycin complex (mTORC). Phosphatase and tensin homolog (PTEN), a tumor suppressor, interferes with Akt activation by converting PIP3 to PIP2. The phosphorylation of Akt at residue S473 either by mTOR or by deoxyribonucleic acid (DNA)-dependent protein kinase (DNA-PK) induces its full activation. This leads to additional substrate-specific phosphorylation, including the inhibitory phosphorylation of the pro-apoptotic Forkhead box O (FOXO). Overall, the PI3K/AKT pathway induces cell survival, cycle progression, and cell growth. 

Targeting the PI3K/Akt pathway in castration-resistant prostate cancer

The importance of this pathway in CRPC pathogenesis is highlighted by genomic studies that have found its deregulation in 42% and 100% of localized and advanced-stage prostate cancer, respectively [7]. Supportive evidence came from the mutation of pathway inhibitors like PTEN. It has been shown that castration-resistant growth is an intrinsic property of PTEN null prostate cancer [8].

The fundamental findings have made this pathway a therapeutic target in clinical trials for CPRC. These trials have been reviewed by Bitting and Armstrong [9]. According to their review, the PI3K/Akt pathway inhibitors target the main actors: PI3K, Akt, or mTOR, and some of them have PI3K/mTOR dual inhibition. These inhibitors have generally an acceptable safety profile, with common side effects including nausea, vomiting, diarrhea, and hyperglycemia. Inhibitors of PI3K or Akt seem to be more effective than mTOR inhibitors; this fact may be due to the incomplete inhibition of the pathway by the latter. Unfortunately, these inhibitors have not met expectations as a monotherapy. For this reason, researchers have combined them with other therapeutics such as antiandrogens, chemotherapy, and mitogen-activated protein kinase (MEK) inhibitors. The focus in this paper will be on the PI3K/Akt-androgen dual blockade.

PI3K/Akt inhibitors-antiandrogen combination

CPRC are defined by their refractory behavior to androgen inhibitors. Adding an antiandrogen in this condition seems to be counterintuitive. But the rationale comes from findings that this resistance may be reversible and is due in some cases to Akt activation [10]. The inhibition of the Akt pathway is supposed to restore androgen response, especially in PTEN mutant cells, and may require the addition of androgen inhibitors [10]. This suggestion was confirmed in experimental model cells, as reported by previous researchers [10].

However, clinical trial results are variable [9]. Possible explanations reported by the researchers are the difference in the degree of blockade of the PI3/Akt pathway by the inhibitors and the inadequacy of certain antiandrogens used as bicalutamide. The optimal combination would be either a PI3K or an Akt inhibitor with a new generation antiandrogen. In fact, some trials are already using this strategy, citing among them the association of abiraterone acetate (new generation antiandrogen) with either buparlisib (pan PI3K inhibitor), dactolisib (dual pan-PI3K/mTOR inhibitor), or ipatasertib (Akt inhibitor) [11-12]. The results are encouraging, notably in carriers of PTEN mutation [12]. But how could this association be optimized? One possible way is the addition of a third component, but which molecule to use? Given the encouraging clinical data of metformin use in CPRC [13], and other reasons detailed below, the suggestion that the addition of metformin to the PI3K/Akt-antiandrogen dual blockade would be advantageous is made. This hypothesis will be developed in the next section. 

PIP3/Akt inhibitors-antiandrogens-metformin trio

Among the principal arguments advocating the inclusion of metformin are the safety profile, its synergism with the two other compounds, PI3K/Akt inhibitors and antiandrogens, and its therapeutic effect on PIP3/AKT inhibitors common side effects.

Metformin Safety Profile

The well-established safety profile of metformin renders the risk of additional unexpected toxicity less probable. This feature is important, notably in the setting where newer drugs are used concomitantly, as in the case of the PI3K/AKT-antiandrogen dual blockade.

Metformin - PI3K/Akt Synergism

The inhibition of mTOR: mTOR is the main target of pi3K /Akt inhibitors, but it is also inhibited by metformin via AMP-activated protein kinase (AMPK) activation [14].

FOXO3a activation: FOXO3a is a member of the Forkhead box (FOX) superfamily transcription factors. Its inactivation has been implicated in the progression of prostate cancer towards castration resistance, making its activation a therapeutic option [15]. As mentioned earlier, the full activation of Akt induces FOXO3a inactivation, meaning that PI3K/Akt inhibitors would activate FOXO3a. This expectation was confirmed by Das and collaborators [16]. As for metformin, Eveline et al. have demonstrated its role in FOXO3a activation via AMPK [17].

FOXM1 inhibition: FOXM1 is also a member of the Forkhead box (FOX) superfamily transcription factors. In contrast to FOXO3a, FOXM1 is implicated in the pathogenesis of prostate cancer [18]. Moreover, Ketola et al. highlighted its role as the central driver of the most aggressive PCS1 subtype of CRPC [19]. FOXM1 is upregulated by the Akt pathway, making it a target of Akt inhibitors [20]. It is also inactivated by metformin, possibly via AMPK [21-22].

Cyclin D1: PI3K/Akt inhibitors and metformin have both been shown to reduce the level of cyclin D1, an inducer of cell cycle progression and prostate cancer cell proliferation [23-24].

Metformin - Antiandrogens Synergism

De novo fatty acid synthesis: As reported by Xiang et al., de novo fatty acid synthesis occurs at a very low rate in normal cells but it accounts for about 90% of fatty acid formation in prostate cancer cells [25]. Thus, it is an important process for their growth. It is induced by androgen and inactivated by AMPK, making it a target for both anti-androgen therapy and AMPK activators [25]. Metformin, as an AMPK activator, may have this effect, thereby enhancing antiandrogen efficacity.

Antiandrogen resistance: Metformin has been reported to reverse prostate cancer cells' resistance to the new generation antiandrogen enzalutamide [26].

IGF1 receptor expression: The androgen-induced insulin-like growth factor-1 (IGF1) receptor expression in prostate cancer cells is implicated in the proliferation and invasiveness of these cells [26]. Metformin suppresses this induced expression of the IGF1 receptor and thus expands the activity of androgen inhibitors [27].

Metformin, The Treatment of a PI3K /AKT Inhibitors Side Effect

Metformin is the first-line drug for the management of sustained grade 1, grade 2, and asymptomatic grade 3 hyperglycemia [28]. In a retrospective study by Khan et al., 20 among 22 patients who needed medication for hyperglycemia induced by AKT/PI3K inhibitors were treated exclusively by metformin [29].

The above arguments make metformin regarded as a valuable addition to the PI3K/Akt-antiandrogen dual blockade. However, theoretically, there is a risk of early withdrawal, as metformin and PI3K/Akt inhibitors share a common side effect: diarrhea. Characteristically, it occurs soon after the start of metformin or after a dosage increase, although late-onset diarrhea is also reported [30]. An aggravation of this side effect upon the introduction of metformin to patients taking PI3K/Akt inhibitors would be expected, leading to drug discontinuation. Practically, the retrospective study by Khan et al. had not reported this event [29]. However, prospective studies for metformin inclusion should follow some precautions to increase its tolerability such as slow titration of the dosage and taking it with meals [30].

## Conclusions

Including commonly-used medications with a well-established safety profile in cancer treatment based on solid pharmacologic backgrounds is an attractive idea. This is the case of metformin addition to the PI3K/Akt-antiandrogens dual inhibition therapeutic scheme in castration-resistant prostate cancer. Although this is a reasonable hypothesis, clinical trials are needed to evaluate the outcomes of this therapeutic combination.
